# Knowledge, attitudes, and practices regarding intranasal corticosteroid use in adult outpatients with allergic rhinitis: a cross-sectional study in Sichuan Province, China

**DOI:** 10.3389/fpubh.2026.1815117

**Published:** 2026-05-12

**Authors:** Hongting Zhang, Xia Li, Tianyi Guo, Huiling Zhao

**Affiliations:** 1 Department of Otolaryngology-Head and Neck Surgery, West China Hospital, Sichuan University/West China School of Nursing, Sichuan University, Chengdu, China; 2 West China School of Nursing, Sichuan University, Chengdu, China

**Keywords:** allergic rhinitis, intranasal corticosteroids, knowledge, attitudes, practices, medication adherence, patient education

## Abstract

**Objective:**

This study aimed to investigate the Knowledge, Attitudes, and Practices (KAP) regarding Intranasal Corticosteroid (INCS) use among adult patients with allergic rhinitis (AR) in Sichuan Province, China.

**Methods:**

A cross-sectional study was conducted using an online questionnaire distributed to 262 adult AR outpatients recruited from a tertiary hospital in Sichuan Province, China. The survey assessed demographic characteristics, nasal symptoms, and KAP related to INCS use.

**Results:**

While most patients (88.93%) recognized INCS as an effective AR treatment, nearly half (49.24%) held misconceptions about systemic side effects from long-term use. Significant knowledge gaps were identified in administration techniques, with less than 40% correctly identifying proper head position (38.17%) or nozzle direction (39.31%). A notable intention-implementation gap was observed between positive attitudes and actual practices. Attitudes were generally positive, especially toward treatment adherence and receiving professional guidance (7.47/10). However, practice adherence was suboptimal: only about 65% consistently followed prescribed dosage/frequency, and performance was poorer for technically demanding steps like using the contralateral hand (50.8%) and directing spray laterally (43.9%).

**Conclusion:**

Despite positive attitudes toward INCS, significant knowledge deficits and poor practice adherence persist, with common safety misconceptions and inadequate technique mastery observed in this sample. Given the cross-sectional design and lack of confounder modelling, these findings are descriptive and hypothesis-generating. Skills-based education incorporating hands-on teach-back may be considered, but its impact should be evaluated in future multivariable and prospective/interventional studies.

## Introduction

Allergic rhinitis (AR) is a chronic inflammatory condition of the nasal mucosa, characterized by symptoms such as nasal congestion, episodic sneezing, rhinorrhea, and nasal itching (1). Evidence indicates that AR affects approximately 5 to 40% of the global population, with its prevalence demonstrating a consistent upward trend in recent years (2).

According to clinical guidelines, intranasal corticosteroids (INCS) are recommended as the first-line treatment for adults and children aged 2 years and older with persistent or moderate-to-severe allergic rhinitis, due to their established high efficacy (3–5). However, survey results reveal that nearly half of the patients perceive the medication’s efficacy as insufficient (6). Evidence indicates that incorrect INCS self-administration is common, and suboptimal technique is associated with reduced pharmacological efficacy, more local adverse effects, and poorer adherence (7). This lack of knowledge often leads to confusion and resistance toward INCS, consequently resulting in suboptimal adherence and compliance. Poor adherence is a major barrier to achieving control of AR (8). Importantly, inadequate INCS use is not limited to missed doses. Inaccurate administration technique (e.g., head position and spray direction, particularly aiming toward the septum instead of slightly outward) may compromise effective intranasal delivery and tolerability, thereby reinforcing nonadherence (9, 10).

Studies report that INCS use in AR is frequently suboptimal, with adherence typically around the mid-range rather than optimal, and common obstacles including symptom improvement leading to intermittent use, forgetfulness, and concerns about adverse effects (11, 12). In addition, patient instruction and mastery of correct spray technique are often insufficient, and technique errors remain common in routine practice (9). Despite the growing number of reports, current international evidence still has several limitations, including variation in study settings and outcome definitions, reliance on self-reported use or locally developed measures, and insufficient KAP domain-level analyses to explain nonadherence and misuse (11, 12).

The Knowledge, Attitude, and Practice (KAP) model refers to the process through which individuals, based on acquired knowledge, form a positive attitude that ultimately promotes behavioral change. As the recipients of medications, patients’ knowledge, attitudes, and behaviors play a pivotal role in determining treatment outcomes (13). Therefore, assessing patients’ current KAP status regarding INCS and identifying influencing factors are essential for improving medication use efficiency. Although KAP-related investigations on AR and INCS have been reported in different settings (14), evidence remains limited on INCS-specific KAP within mainland Chinese adult patients. In particular, patients’ knowledge and practice of correct INCS administration technique and their concerns about steroid safety have not been sufficiently described (7, 15). Moreover, KAP surveys are often descriptive and may not explain why favorable attitudes do not translate into correct, sustained real-world use. To strengthen interpretability and derive actionable education strategies, we adopted the Capability Opportunity Motivation Behaviour (COM-B) model (16). Under this framework, knowledge deficits primarily reflect psychological capability, technique errors reflect physical capability (skills), safety concerns reflect reflective motivation, and real-world implementation is also shaped by opportunity (e.g., access to instruction, cues, and follow-up support).

This cross-sectional study aims to investigate the knowledge, attitudes, and practices (KAP) of patients with allergic rhinitis (AR) regarding intranasal corticosteroids (INCS). The findings will identify key gaps and misconceptions that may inform the development of targeted patient education programs, thereby enhancing adherence and optimizing treatment outcomes in this population.

## Methods

This cross-sectional study employed online questionnaires to investigate KAP regarding INCS use among adult patients with AR. This study was conducted among adult outpatients in Sichuan Province, Southwest China (West China Hospital, Chengdu), to reflect routine AR care in a high-volume tertiary setting serving both urban and rural populations. Data were collected from May to September 2025. This timeframe was selected to ensure a stable outpatient caseload and to capture routine clinical presentations of AR in real-world practice during the study period. Ethical clearance was obtained from the West China Hospital Institutional Review Board (Approval No. 2025-671). Participation was voluntary, and electronic informed consent was obtained prior to questionnaire initiation. All responses were anonymous, and data were stored on password-protected devices accessible only to the research team.

### Questionnaire

A multidisciplinary expert panel developed the survey to assess knowledge, attitudes, and practices (KAP) regarding the use of INCS in Patients with allergic rhinitis. Item generation was informed by previously validated KAP-INCS instruments, and was expanded to capture technique-critical domains (e.g., head position and spray direction) relevant to real-world INCS effectiveness (8).

The questionnaire comprises five domains: (1) demographic characteristics (8 items assessed sex, age, educational level, severity of allergic rhinitis, presence of other allergic comorbidities, family history of allergic rhinitis, types of allergens, and whether they had received guidance on the use of INCS); (2) allergic rhinitis nasal symptom severity (4-item Visual Analog Scale); (3) INCS knowledge (7 items evaluating pharmacological understanding and administration techniques); (4) attitudinal measures(7-item VAS scales assessing Attitude toward INCS Usage); and (5) administration practices (7-item Likert scale evaluating proper INCS usage methods). To ensure standardization and reproducibility, the finalized questionnaire and scoring rubric are provided in Supplementary Appendix S1.

Prior to data collection, the preliminary questionnaire underwent independent evaluation by a multidisciplinary review panel comprising medical educators, allergists, and specialized nurses. Incorporating their expert feedback, we refined the survey items for clarity and clinical relevance. The revised version was then pilot-tested with 15 parents of children with allergic rhinitis to assess the comprehensibility of the instructions and the accuracy of the terminology. Following this validation process, the research team finalized the instrument for deployment. Finally the scale showed strong psychometric properties in this study. High internal consistency supported reliability: Cronbach’s alpha was 0.953 (attitude) and 0.808 (practice). Validity assessment was feasible. The Kaiser–Meyer–Olkin (KMO) values were 0.889 (attitude) and 0.701 (practice) (both *p* < 0.01), confirming that the scale exhibits good reliability and validity.

### Participants

A convenience sampling method was employed to survey patients with allergic rhinitis who visited the outpatient department of a tertiary hospital in Sichuan Province. To ensure a standardized diagnostic reference, allergic rhinitis (AR) was confirmed by the treating otolaryngologist/allergist according to international guidance (ICAR), based on (1) a compatible clinical history of typical nasal symptoms (e.g., sneezing, rhinorrhea, nasal obstruction/congestion, and/or nasal itching) and (2) objective evidence of aeroallergen sensitization (positive skin-prick test and/or serum allergen-specific IgE) documented in the medical record. Patients without documented sensitization evidence or with an alternative rhinitis diagnosis were not eligible (17).

Eligible participants met all of the following criteria: (1) age ≥18 years; (2) clinician-confirmed AR as defined above; and (3) current or recent (within the past 4 weeks) use of an intranasal corticosteroid prescribed for allergic rhinitis. Patients were excluded if they had non-allergic rhinitis, acute upper respiratory tract infection within the preceding 2 weeks, chronic rhinosinusitis with or without nasal polyps, or any condition that could preclude independent questionnaire completion (e.g., cognitive impairment). Participation was voluntary, with consent obtained through responses to the initial survey question; those who declined were excluded.

Of the 265 questionnaires distributed, 262 (98.87%) met the criteria for subsequent data analysis. During the filtration process, two respondents were removed for withdrawing their consent.

### Theoretical framework

The study was anchored in the COM-B model, which conceptualizes health behaviours as arising from an interaction between Capability, Opportunity, and Motivation (16). Knowledge items were interpreted as psychological capability (understanding of indications, safety, and administration steps), technique-related practice items as physical capability (skills for correct use), and attitude items as reflective motivation (beliefs about necessity, concerns, and willingness to use INCS as recommended). Opportunity was not directly quantified by a dedicated scale in this survey; therefore, opportunity-related explanations were restricted to the care context and the feasibility of providing instruction, cues, and follow-up checks. This framework was used to organise the presentation of results and to derive education-oriented recommendations aligned with known behaviour-change techniques (e.g., instruction, demonstration, teach-back, action planning, and practical support).

### Data collection

Following standardized training for the investigators, data were collected using a web-based survey platform. Participants were approached consecutively after their outpatient visit, received a brief standardized explanation of the study, and completed the questionnaire independently via a QR code/link. After reading the study information, participants provided electronic informed consent and completed the questionnaire independently. Eligibility screening items and core KAP items were set as mandatory fields to minimize missing critical data, only one submission per IP address was allowed. The survey platform automatically recorded submission timestamps and completion duration. Questionnaires with a completion time of <3 min were excluded as invalid. To protect privacy, the questionnaire did not collect direct personal identifiers (e.g., name, national ID number, phone number, or exact home address). Datasets was restricted to the research team via password-protected credentials. Data were downloaded for analysis and stored on password-protected devices, and results were reported in aggregate to avoid re-identification.

### Data analysis

Data were analyzed using IBM SPSS Statistics (version 25.0). Descriptive statistics were used to summarize participant characteristics and KAP outcomes. Continuous variables were presented as mean ± standard deviation (SD) or median (interquartile range, IQR), as appropriate, and categorical variables were presented as frequencies and percentages. Normality of continuous variables was assessed (e.g., Shapiro–Wilk test and visual inspection). Group comparisons were conducted using independent-samples *t*-tests (or Mann–Whitney *U* tests for non-normally distributed data) for continuous variables and *χ*^2^ tests (or Fisher’s exact tests, as appropriate) for categorical variables. A two-sided *p*-value of less than 0.05 was considered statistically significant.

## Results

The mean age of the patients was 33.00 years (SD = 9.87). Of the 262 participants, 118 (45.04%) were male and 144 (55.96%) were female. The majority of the patients (*n* = 223, 85.11%) had attained an education level of college or above. Most patients were married (*n* = 158, 60.31%), and over half of the households reported a monthly income exceeding ¥10,000 RMB (*n* = 138, 52.67%). Over one-third of the patients (*n* = 97, 37.02%) were diagnosed with persistent moderate-to-severe rhinitis (Table 1).

**Table 1:** Baseline demographic and clinical characteristics of the study participants (*N* = 262).

Characteristics		*n* (%)
Sex	Male	118 (45.04)
	Female	144 (55.96)
Age (years), mean ± SD	33.00 ± 9.78	
Educational level	Primary school or below	3 (1.15)
	Middle school	13 (4.96)
	High school	23 (8.78)
	College	174 (66.41)
	Postgraduate and above	49 (18.70)
Marital status	Married	158 (60.31)
	Other	104 (39.69)
Monthly household income	≤5,000 RMB	38 (14.50)
	5,001–10,000 RMB	86 (32.82)
	10,001–20,000 RMB	81 (30.92)
	Above 20,000 RMB	57 (21.76)
Disease severity	Intermittent-mild	44 (16.79)
	Intermittent-moderate-severe	81 (30.92)
	Persistent-mild	40 (15.27)
	Persistent-moderate–severe	97 (37.02)

Based on the 2025 Sichuan per capita disposable income of 36,120 RMB/year (~3,010 RMB/month), a typical three-person household averages approximately 10,000 RMB/month. Accordingly, household monthly income of <10,000 RMB is below average, 10,000–30,000 RMB is middle-income, and >30,000 RMB is upper-middle to high income.

### Information on allergic rhinitis symptoms, subtypes, and allergens

Among the patients, 107 cases (40.84%) had other family members with allergic rhinitis. Regarding comorbidities, 45.42% (*n* = 119) of patients with allergic rhinitis presented with other allergic diseases, with asthma being the most common (*n* = 62, 23.66%), followed by atopic eczema (*n* = 23, 8.78%).

In terms of allergen distribution, house dust mites constituted the most prevalent allergen (*n* = 176, 70.97%), followed by spring pollens (e.g., Juniperus, Paper Mulberry, and Phoenix Tree) at 47.98% (*n* = 119) and autumn pollens (e.g., *Humulus scandens*, *Artemisia annua*, and Artemisia sieversiana) at 22.58% (*n* = 56).

Regarding AR classification, the persistent moderate-to-severe and intermittent mild types showed the highest prevalence at 37.02% (*n* = 97) and 30.92% (*n* = 81), respectively. The severity of allergic rhinitis symptoms (sneezing, rhinorrhea, nasal itching, and nasal congestion) was assessed using a Visual Analog Scale (VAS). Overall, symptoms were rated as moderate. Nasal congestion/obstruction had a median VAS score of 4.0 (IQR 2.0–7.0), compared with 4.0 (IQR 2.0–6.0) for paroxysmal sneezing, clear rhinorrhea, and nasal pruritus (Table 2).

**Table 2:** VAS scores for nasal symptoms in patients with allergic rhinitis (*N* = 262).

Items	MD (*Q*_25_, *Q*_75_)
Severity of paroxysmal sneezing episodes (≥3 times/day)?	4.0 (2.0–6.0)
Severity of clear rhinorrhea (watery nasal discharge) per day?	4.0 (2.0–6.0)
Severity of nasal pruritus (itching) per day?	4.0 (2.0–6.0)
Severity of nasal congestion/obstruction per day?	4.0 (2.0–7.0)

### Knowledge of the usage of INCS

**Figure 1:**
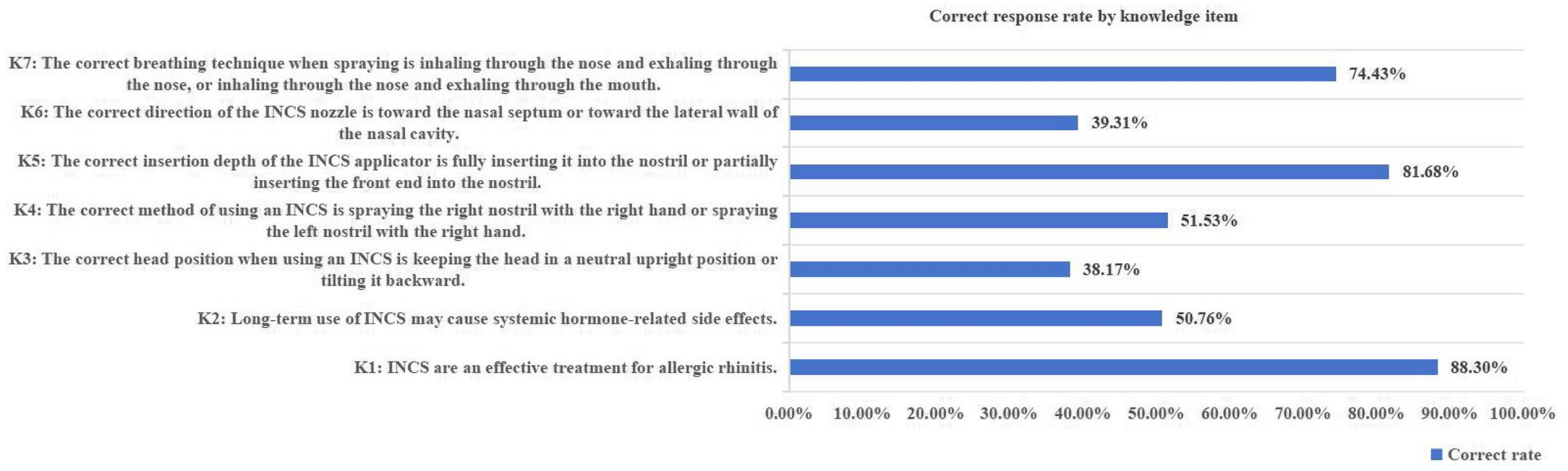
Correct response rates for INCS-related knowledge items (*N* = 262). The corresponding questionnaire items are provided in Supplementary Appendix S1.

In the knowledge category, while awareness of general treatment principles was high, understanding of correct practical techniques was critically deficient. Regarding fundamental knowledge, a large majority of participants (88.93%, *n* = 233) correctly recognized INCS as an effective treatment for allergic rhinitis. However, only half of the respondents (50.76%, *n* = 133) accurately understood that long-term use of INCS is not typically associated with systemic hormone-related side effects. Concerning administration techniques, the majority of participants (81.68%, *n* = 214) correctly understood that partial insertion of the tip into the nostril represents proper insertion depth, and proper breathing technique (inhaling through the nose while exhaling through the mouth) was identified by 74.43% (*n* = 195). However, the correct response rates for proper head position and appropriate nozzle direction were both below 50%, specifically 38.17% (*n* = 100) and 39.31% (*n* = 103), respectively (Figure 1)

### Attitude toward the use of INCS

We assessed attitudes toward INCS using a VAS. Participants demonstrated generally positive attitudes regarding intranasal corticosteroid use across multiple domains (Table 3). The highest scores were observed for items related to treatment adherence, with completing the full course of treatment (7.71 ± 2.35) and using INCS at the prescribed daily frequency (7.67 ± 2.41) receiving the strongest agreement. Participants also placed considerable importance on proper administration techniques, showing strong belief in using the correct dosage (7.52 ± 2.38) and employing proper spraying technique (7.46 ± 2.46). Furthermore, respondents expressed substantial motivation for education, demonstrating strong interest in receiving professional guidance from healthcare providers (7.47 ± 2.60) and learning more INCS-related knowledge (7.02 ± 2.72). The general importance of INCS in allergic rhinitis management received a comparatively lower, though still positive, rating (6.46 ± 2.63). Notably, female patients demonstrated significantly higher total attitude scores toward INCS use compared to male patients (53.87 ± 14.35 vs. 48.19 ± 16.39, *t* = −2.99, *p* = 0.003).

**Table 3:** Attitudes toward the use of INCS (*N* = 262).

Attitudes questionnaire	Score (M ± SD)
1. Using INCS is important for the treatment of allergic rhinitis.	6.46 ± 2.63
2. I would like to learn more about INCS-related knowledge.	7.02 ± 2.72
3. I hope to receive guidance from healthcare professionals on the correct use of INCS.	7.47 ± 2.60
4. The correct INCS technique is important for the therapeutic effectiveness in allergic rhinitis.	7.46 ± 2.46
5. Using the correct dosage of INCS each time is important for the therapeutic effectiveness in allergic rhinitis.	7.52 ± 2.38
6. Using INCS at the prescribed frequency every day is important for the therapeutic effectiveness in allergic rhinitis.	7.67 ± 2.41
7. Completing the full course of INCS treatment as prescribed is important for the therapeutic effectiveness in allergic rhinitis.	7.71 ± 2.35

### Practice on the use of INCS

The evaluation of INCS administration practices revealed moderate adherence to prescribed medication regimens, with 64.9% (always/often) of participants consistently following the correct dosage and 64.5% (always/often) adhering to the recommended frequency. In terms of administration techniques, the proper nozzle insertion depth was implemented by 61.0% of respondents (always/often). However, performance of more technically demanding maneuvers was notably poorer: only 50.8% (always/often) of participants consistently used the contralateral hand technique, and correct lateral nozzle direction was maintained by merely 43.9% (always/often) (Table 4).

**Table 4:** Self-reported practices regarding INCS use (*N* = 262).

Items	Always *N* (%)	Often *N* (%)	Sometimes *N* (%)	Occasionally *N* (%)	Never *N* (%)
1. I use INCS strictly according to the dosage prescribed by my physician.	87 (33.21)	83 (31.68)	62 (23.66)	25 (9.54)	5 (1.91)
2. I use INCS strictly according to the frequency prescribed by my physician.	82 (31.30)	87 (33.21)	63 (24.05)	24 (9.16)	6 (2.29)
3. I maintain a neutral, upright head position when using INCS.	85 (32.44)	86 (32.82)	60 (22.90)	23 (8.78)	8 (3.05)
4. I partially insert the nozzle tip into the nostril during INCS administration.	91 (34.73)	69 (26.34)	59 (22.52)	26 (9.92)	17 (6.49)
5. I use the contralateral hand technique (e.g., right hand to left nostril) when using INCS.	71 (27.10)	62 (23.66)	60 (22.90)	28 (10.69)	41 (15.65)
6. I direct the spray toward the lateral wall of the nasal cavity when using INCS.	62 (23.66)	53 (20.23)	59 (22.52)	42 (16.03)	46 (17.56)
7. I coordinate spray activation with gentle nasal inhalation followed by oral exhalation.	79 (30.15)	73 (27.86)	64 (24.43)	28 (10.69)	18 (6.87)

## Discussion

This cross-sectional study describes the KAP regarding INCS use among patients with allergic rhinitis, revealing a significant divergence between patient attitudes and their actual knowledge and practices. While participants demonstrated generally positive attitudes toward treatment adherence and a strong motivation for education, these favorable dispositions were insufficient to ensure optimal medication use. Three findings from our results illustrate this gap: (1) only 64.9% consistently used the correct dosage, (2) only 50.8% used the contralateral hand technique, and (3) only 43.9% directed the spray laterally. The central challenge identified was not merely a knowledge-practice gap but, more critically, an intention-implementation gap, wherein patients who understood the correct techniques frequently failed to execute them consistently. These gaps may be related to critical deficits in two domains: misconceptions regarding INCS safety and inadequate mastery of administration techniques.

Regarding general knowledge and safety perceptions, our findings delineate a notable disparity between the recognition of INCS efficacy and misconceptions surrounding their safety profile. A substantial majority (88.93%) of patients correctly identified INCS as an effective first-line treatment for AR, a proportion exceeding the 66.2% reported in a comparable study (6). This heightened awareness reflects the well-established role of INCS in clinical guidelines. However, this informed perspective on efficacy was juxtaposed with prevalent safety concerns, as nearly half of the cohort (49.24%) erroneously associated long-term INCS use with systemic hormone-related side effects—a misconception aligning with prior observations of “steroid phobia” (6, 9). This divergence is clinically critical, given that modern INCS are characterized by minimal systemic bioavailability and a well-documented favorable long-term safety record (10). However, given the cross-sectional design and the lack of multivariable adjustment, the present study cannot determine whether such concerns independently explain nonadherence or incorrect use. This finding highlights the pivotal role of healthcare professionals in proactively addressing safety concerns during clinical consultations.

Our results further identified pronounced knowledge deficits in administration technique, consistent with previous reports that a majority of patients possess little or no knowledge about correct INCS use (14, 15, 18). However, in contrast to the findings of Gu et al. (15), who reported a positive correlation between knowledge level and educational attainment, our study found that over 66% of respondents had a college degree or higher, yet their knowledge of relevant techniques remained poor. The possible reason for this discrepancy is that general education rarely covers the operational details of specialized medication use, and such knowledge often relies on professional training or direct guidance from healthcare providers rather than on common-sense reasoning. This distinction aligns with the COM-B framework, which differentiates general capability (e.g., educational attainment) from specific physical capability (e.g., task-specific medication administration skills) (16). These deficits were particularly evident in technically demanding maneuvers; for instance, less than 40% of respondents identified the correct head position or nozzle direction. Improper techniques, such as incorrect head tilt or directing the spray toward the nasal septum, fundamentally impair drug deposition onto the target nasal mucosa. This not only diminishes the intended clinical benefit—potentially leading to the erroneous conclusion of treatment failure—but may also increase the risk of local adverse effects like epistaxis (19). Consequently, the mere act of prescribing is insufficient; clinicians may consider complementing it with structured, hands-on training using demonstration and “teach-back” methods to verify technique competency, although the effectiveness of such strategies should be evaluated in prospective or interventional studies.

Despite these technical knowledge gaps, our study found that patients held generally positive attitudes toward the importance of standardized treatment regimens and proper technique, corroborating the findings of Retinasekharan et al. (6). Participants also exhibited substantial motivation for education, as reflected by a strong desire for professional guidance (score: 7.47/10). This provides a favorable foundation for implementing targeted educational interventions. Notably, sex emerged as a significant factor influencing attitudes, with female patients reporting significantly higher attitude scores than their male counterparts. This observed disparity underscores the importance of considering sex-specific communication and support strategies in clinical practice.

However, these positive attitudes did not reliably translate into optimal practices, revealing a substantial intention-implementation gap. While medication adherence was moderate—with approximately 65% of patients consistently adhering to the prescribed dosage and frequency, a figure aligned with prior research (6)—the execution of administration techniques was notably poorer. This gap is vividly illustrated by the dissociation between knowledge and practice concerning the correct breathing technique: while 74.43% of participants knew the method, only 58.01% reported consistently executing it. The performance was even lower for more complex maneuvers, with only 50.8% consistently using the contralateral hand technique and a mere 43.9% consistently directing the spray toward the lateral nasal wall. These technical shortcomings, consistent with earlier studies (19, 20), collectively underscore that inadequate administration technique remains a widespread and persistent issue.

It is noteworthy that these technical deficiencies persisted despite our cohort’s high educational attainment, suggesting that the challenge transcends information accessibility. The core issue lies in translating abstract knowledge into proficient, habitual motor skills—a process that requires repeated, hands-on coaching and reinforcement, which is often absent in standard clinical encounters. The multi-step administration process is inherently complex, and unsupervised daily use readily allows improper habits to form and solidify. To bridge this gap, clinicians must move beyond verbal instruction or brief demonstration. We recommend a structured educational approach that includes: (1) physically demonstrating the correct technique; (2) concurrently explaining the underlying rationale (e.g., how gentle nasal inhalation optimizes drug deposition while oral exhalation minimizes pulmonary exposure) to foster deeper comprehension; and (3) implementing a mandatory “teach-back” assessment wherein patients demonstrate the technique back to the clinician. This allows for the immediate identification and correction of error-prone steps, thereby verifying competency and ensuring proper long-term administration. However, these recommendations require confirmation in rigorously designed prospective or interventional studies. Beyond the content of education, the context in which education is delivered may also matter. Furthermore, Al-Rasheedi et al. (14) reported that follow-up settings were significantly associated with patients’ knowledge and practice, with patients followed up at pharmacies exhibiting significantly poorer practices (*p* = 0.03), indicating that future interventional studies are warranted to explore the impact of different follow-up models on patients’ knowledge and practice.

In the study, the sample was highly educated, with 85.11% reporting a college-level education or above. This distribution likely reflects the tertiary-care outpatient setting and the online survey mode, which may preferentially recruit individuals with higher educational attainment and better digital access, thereby limiting representativeness. Web-based surveys can yield systematically different estimates from other modes due to undercoverage and self-selection, and differences may persist even after demographic adjustment (21). However, substantial gaps remained in technique-critical knowledge (e.g., head position and nozzle direction) and corresponding real-world practices even in this relatively well-educated sample. This suggests that higher educational attainment alone may not ensure correct INCS use, and skills-based patient education remains necessary in routine care. Future studies should include more diverse educational backgrounds to examine whether KAP gaps differ across subgroups.

## Limitation

The generalizability of our findings may be limited by the use of a convenience sample recruited from a single tertiary hospital. This sample, characterized by its high educational attainment, a predominance of moderate-to-severe AR and access to superior healthcare resources at a high-level hospital, may not be representative of the general AR patient population, thus potentially leading to an overestimation of the overall KAP levels among AR patients. In addition, KAP measures were self-reported and may be subject to recall and social desirability biases, potentially inflating reported attitudes or adherence. Because the study was purely descriptive, cross-sectional and did not apply multivariable adjustment, causal inference is not supported and residual confounding may remain. Future multi-center studies with more diverse samples and more rigorous analytical approaches are warranted. Additionally, we did not assess access, affordability, or healthcare setting (e.g., primary care vs. pharmacy follow-up), which may influence correct INCS usage. Future studies must incorporate these factors to improve understanding and outcomes.

## Conclusion

This study identified a gap between favorable attitudes toward intranasal corticosteroids and suboptimal knowledge and practice among adults with allergic rhinitis. Misconceptions about long-term safety (49.24%) and administration technique deficiencies were common (e.g., only 38.2 and 39.3% correct for head position and nozzle direction, respectively) and may contribute to inappropriate real-world use. Given the cross-sectional design, these associations should be interpreted cautiously. Targeted, skills-based education with teach-back may be considered as a potential strategy to address technique-related deficits, and future studies should confirm determinants and clinical impact using multivariable and prospective designs.

## Data Availability

The raw data supporting the conclusions of this article will be made available by the authors, without undue reservation.
