# Supplementary material for: Knowledge, attitudes, and practices regarding intranasal corticosteroid use in adult outpatients with allergic rhinitis: a cross-sectional study in Sichuan Province, China
Source: Front Public Health. 2026 May 12;14:1815117. doi: 10.3389/fpubh.2026.1815117 (PMC13201201; doi:10.3389/fpubh.2026.1815117)
Supplement: Supplementary file 1 [file Supplementary_file_1.docx]

### Supplementary Appendix S1

### Survey on Knowledge, Attitudes, and Practices Regarding Intranasal Corticosteroid Use in Adult Outpatients with Allergic Rhinitis

Dear participant,

Thank you for your participation.

This survey asks about your knowledge, attitudes, and daily use of intranasal corticosteroids. Please answer honestly. Your responses are confidential and used only for academic research.

**Would you like to take this survey?**

☐ Yes ☐ No

### Section 1: General Information

**1.Your age:**_____ years.

**2.Your gender:**☐ Male ☐ Female

**3.Your education level:**

☐Primary school or below ☐Middle school ☐High school ☐College ☐ Postgraduate or above

**4. Your occupation:**

☐ Student ☐ Employed ☐ Unemployed ☐ Self-employed

**5. Marital status:**

☐ Unmarried ☐ Married ☐ Divorced ☐ Widowed

**6. Monthly household income (RMB):**

☐ <5000 ☐ 5001–10000 ☐ 10001–20000 ☐ >20000

**7. Payment method:**

☐ Medical insurance ☐ Commercial insurance ☐ Self-paid ☐ Other

**How long have you had allergic rhinitis?** ____ months

**What type of allergic rhinitis do you have?**

☐Intermittent-mild ☐Intermittent-moderate/severe ☐Persistent-mild ☐ Persistent-moderate/severe

Note:Intermittent = symptoms occur <4 days/week or <4 consecutive weeks;

Persistent = symptoms occur 4 or more days each week or for 4 or more consecutive weeks.

Mild = symptoms are tolerable and do not affect normal life;

Moderate/severe = symptoms are strong and interfere with work, school, or everyday activities.

**Do you have any other allergies?**

☐Asthma ☐Atopic dermatitis ☐Eczema ☐Other

**Do any family members have allergic rhinitis?**

☐ Yes ☐ No

**Have you had allergen testing?**

☐ Yes –Testing method: ☐Skin prick test ️Blood test ☐Nasal provocation test

☐ No

**13. Your allergen(s):**

☐ Dust mites ☐ Spring pollen (e.g., juniper, mulberry, sycamore) ☐ Autumn pollen (e.g., humulus, artemisia) ☐ Cat dander ☐ Dog dander ☐ Mold

1. **Do you use oral antihistamines?**

☐ Yes ☐ No

**15. Have you ever received instructions from healthcare professionals on how to use a nasal spray?**

☐ Yes ☐ No

**16. Which nasal spray do you use?**

☐ Mometasone furoate (Nasonex) ☐ Fluticasone propionate (Flixonase) ☐ Budesonide (Rhinocort) ☐ Other

### Section 2: Allergic rhinitis nasal symptom severity

Please rate your symptoms in the past 24 hours. (0 = no symptoms, 10 = very severe)

**17. Severity of sneezing:**


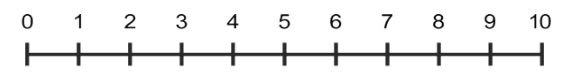


**18. Severity of watery rhinorrhea:**


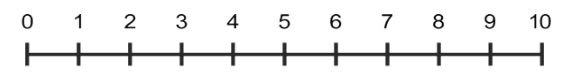


**19. Severity of nasal itching:**


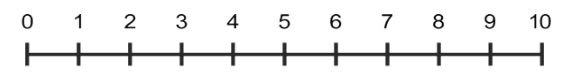


**20. Severity of nasal congestion:**


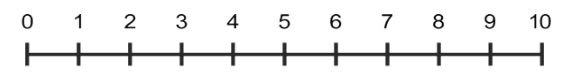


### Section 3:Knowledge, Attitudes, and Practices Regarding Intranasal Corticosteroid Use

### Knowledge

**21. Nasal spray is an effective treatment for allergic rhinitis.**

☐ True ☐ False

**22. Long-term use of a nasal spray is associated with systemic steroid-related side effects.**

☐ True ☐ False

**23. Intranasal corticosteroid sprays contain steroids.**

☐ True ☐ False

**24. What is the correct head position when using a nasal spray?**


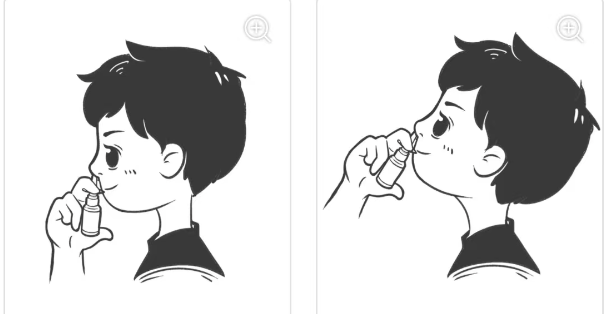


☐ Natural/neutral ☐ Slightly tilted back

**25.What is the correct method for using a nasal spray?**


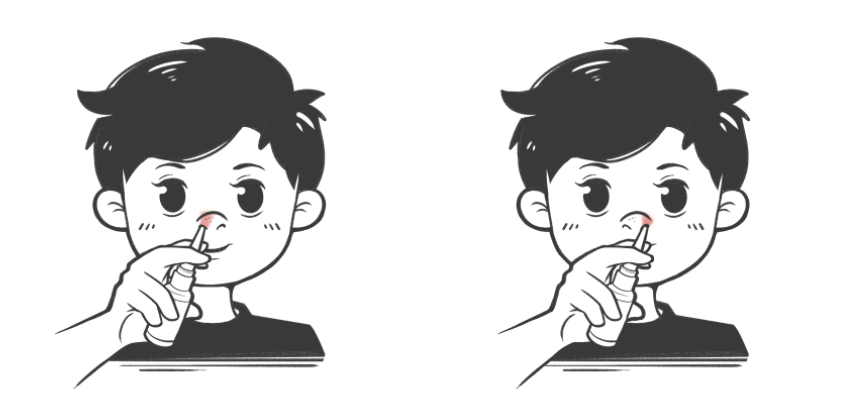


☐ Right hand to right nostril ☐ Right hand to left nostril

**26. What is the correct insertion depth of the nozzle into the nostril?**

☐ Fully inserted ☐ Tip partially inserted

**27.What is the correct direction of the spray nozzle?**


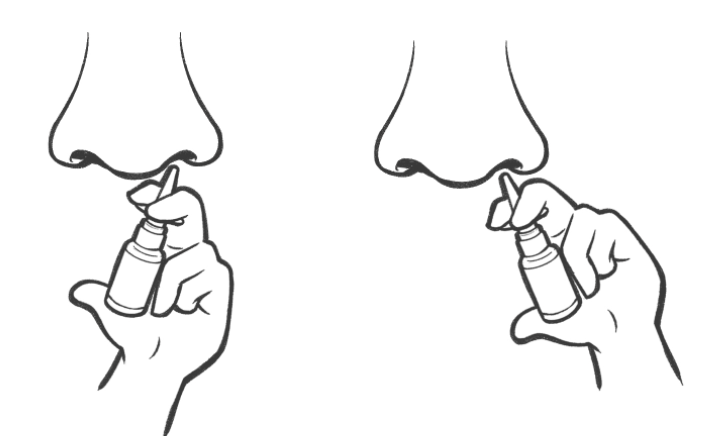


☐ Pointing outward (lateral) ☐ Pointing inward (toward septum).

28.What is the right way to breathe when spraying?


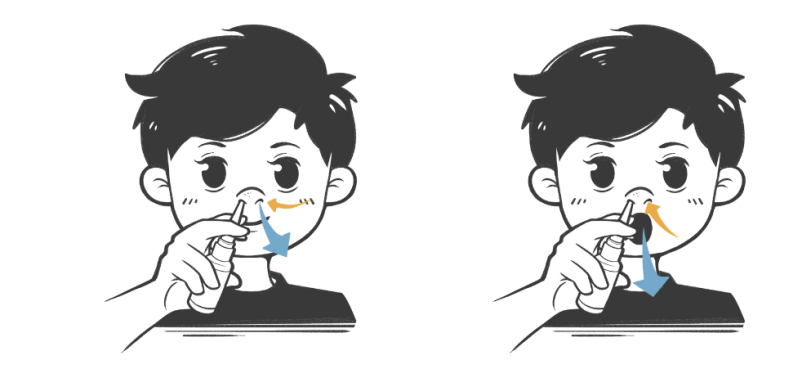


☐Inhale through nose, exhale through nose ☐Inhale through nose, exhale through mouth

### Attitudes

Please rate your agreement (0 = strongly disagree, 10 = strongly agree).*

**29. I believe using a nasal spray is important for treating allergic rhinitis.**


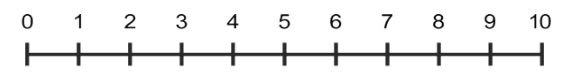


**30. I want to learn more about nasal spray.**


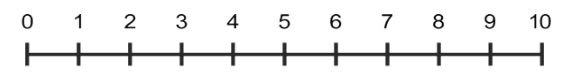


**31. I hope to receive guidance from healthcare professionals on how to use a nasal spray correctly.**


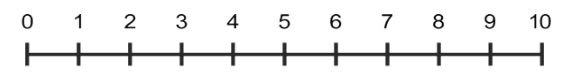


**32. I believe the correct spraying technique is important for treatment effectiveness.**


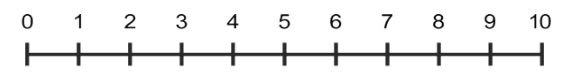


**33. I believe using the correct dosage each time is important for treatment effectiveness.**


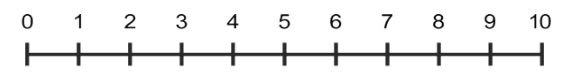


**34. I believe using the prescribed frequency every day is important for treatment effectiveness.**


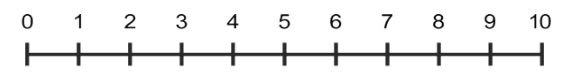


**35. I believe completing the full course of treatment as prescribed is important for treatment effectiveness.**


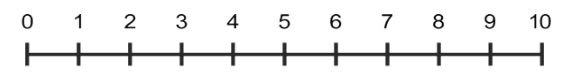


### Practices

For each action, indicate how often you do it.

**36. I use the exact dosage prescribed by my doctor.**

① Never ② Occasionally ③ Sometimes ④ Often ⑤ Always

**37. I use the prescribed frequency every day.**

① Never ② Occasionally ③ Sometimes ④ Often ⑤ Always

**38. I keep a neutral head position when using a nasal spray.**

① Never ② Occasionally ③ Sometimes ④ Often ⑤ Always

**39. I use the contralateral hand technique (e.g., right hand to left nostril).**

① Never ② Occasionally ③ Sometimes ④ Often ⑤ Always

**40. I insert only the tip of the nozzle into my nostril.**

① Never ② Occasionally ③ Sometimes ④ Often ⑤ Always

**41. I direct the spray toward the lateral wall of the nasal cavity.**

① Never ② Occasionally ③ Sometimes ④ Often ⑤ Always

**42.I gently inhale through the nose while spraying, then exhale through the mouth.**

① Never ② Occasionally ③ Sometimes ④ Often ⑤ Always

**Thank you for your participation!**
